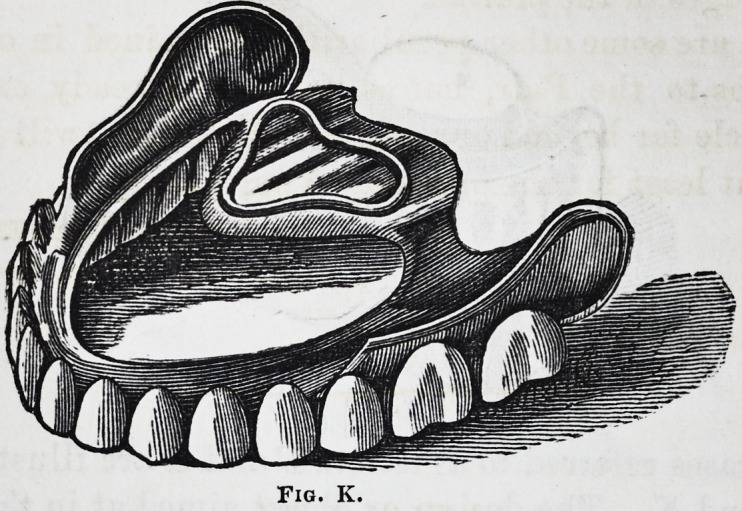# Ambler & Avery's Contributions to the World's Fair

**Published:** 1856-07

**Authors:** 


### ARTICLE V.

### *Ambler & Avery's Contributions to the World's Fair.*

Our professional brethren will understand, and, we trust, appreciate the motives governing us, in thus presenting to them and the public the peculiarities of our contributions to the World's Fair, which have given rise to so much discussion. We do not purpose at this time to renew this discussion, or even allude to it, except as a fact; our object being the presentation of the peculiarities above alluded to, several of which we claim as decided improvements, admitted to be so, and adopted in the practice of some of the most skillful practitioners in the country, (*scientific, practical men.*)

When the proper officers at Washington issued their circular inviting contributions to the exhibition in London, we concluded to send such specimens of practical cases as would illustrate our various methods of mounting and inserting artificial teeth, and made application for space sufficient for said exhibition, which was granted: and in process of time our specimens were upon the walls of the first Crystal Palace; but as is well known to the profession, dentistry or its appliances received no notice whatever at the hands of the jurors or commissioners. Why was this? is a question often asked, but as yet unanswered. Some have assured us that it was an oversight; others have attributed it to an unwillingness to institute a comparison with the European contributions; another reason has been assigned by those who profess to know something of the proceedings behind the curtain, viz: the reception of *certain* letters from *certain* individuals on this side of the Atlantic, asserting that the specimens of mechanical dentistry sent from this country were not made by those who sent them. But be the cause what it may, the fact of their receiving an honorable go-by is notorious.

As we had been at great expense and bestowed much labor on these specimens, and feeling that they combined improvements worthy of commendation, we placed them (as well as several others) on exhibition in the New York Crystal Palace for *competition*, and with them the following letter:

The Judges of Dentistry, &c.—*Gentlemen*:—Permit us to call your attention to the following peculiarities of the specimens contributed by us to the Exhibition, illustrative of our methods of inserting artificial teeth.

We present for your examination these specimens, combining, as we believe, several new, important and practical improvements, viz:

1st.—We claim as our own invention a new method of attaching spiral springs to artificial teeth, as shown in the entire set.

2d.—The principle of producing atmospheric pressure (without central cavities) by raising a bead around the edges and across the arch of plates.

3d.—The peculiar method of mounting or setting blocks, (as illustrated in case marked A,) by screws and steady pins. The advantage of this method you will perceive at first sight; one of which is cleanliness, the blocks can be removed and the plate cleaned at pleasure.

We would also call your attention to the other plates, no two of which are precisely alike. The one marked B, is a lower set of peculiar form, for projecting the lower jaw; the one of which this is a duplicate is worn with entire satisfaction.

The one marked C is a new method of giving weight to the lower teeth, at the same time presenting a gold surface to the mouth.

Cases D, E and F have been worn. D has been worn at intervals by —— (he having another set,) for over three years. E was made for a lady, who wore them (while she lived) with entire satisfaction, and at her death they were returned and received as old gold. F was worn by the wife of ——, who is now dead. The case marked H is a duplicate of one inserted some years since for a gentleman who has lost, not only his teeth, but a portion of the jaw, the cavity extending through and back to the palatine bone. Which loss so affected his speech that it was almost impossible to understand him. The insertion of this plate restored his speech as well as appearance.

You will also see several single teeth lined by melting the gold on them, previously covering the surface of the tooth with platina ribbon and flux. The plate marked G is, as will be seen, inserted on the atmospheric pressure principle, with gum exposed to prevent the diminution of the tasting surface. We have set but few cases in this manner, but they have thus far operated well.

The instruments in the lower part of this case, as you will see, are for beading and banding plates; also a pair of shear-shaped cutting forceps.

We also present our entire case in competition for *workmanship, and as combining a greater variety of styles or methods of setting artificial teeth, &c.,* than any on exhibition.

We would esteem it a privilege to be present at the examination of the specimens and to point out the peculiarities and explain them more fully than we can on paper.

Very respectfully, yours, &c.,

Ambler & Avery, 31 Washington Place.

But as your readers have not the specimens before them, I annex cuts to illustrate some of the peculiarities.

The method of attaching spiral springs (referred to) is represented by the engravings A, B and C. The advantages of this method over the common system of hooking over a bolt are, 1st. You have no projecting bolt to irritate the cheeks. 2d. The springs are less liable to become displaced. 3d. There is no danger of their becoming detached, as that can only be by removing the teeth from the mouth. 4th. The facility for regulating them by stops,

Fig. C.

Figs. A—B.

thereby preventing any irritation or irregularity of action. 5th. The construction is such as to enable the patient to remove or attach them at pleasure, this feature is of more importance than appears at first sight, from the fact that many persons (though successful in the end) find great difficulty at first in wearing full sets of teeth constructed without springs, which difficulty is entirely removed by this method.

Fig. D.

The method of producing atmospheric pressure without central cavities (referred to) is by raising a bead on the inner surface, across the arch and around the edges of the plate, which is done by means of a small machine similar to those used by tin smiths, or a forcep, shaped as in fig. D; the object gained in this method is dispensing with the central cavity, and at the same time securing a vacuum sufficient to cause the plate to adhere to the mouth. The bead or raised portion of the plate imbeds itself in the gum about the 60th portion of an inch; but as the projection is about one-eighth of an inch from the edge of the plate, no irritation is produced, although the bead settles sufficiently to prevent the air from entering under it. This is found to answer a very good purpose and not liable to the same objections as the central cavity.

The method of setting blocks referred to in the above letter, is as follows: (See fig. E.) Having adjusted the

FIG. F. FIG. E.

plate to the mouth, turned the outer band and raised the bead (as above described) across the arch, we make four nuts, (two for the front and one for each of the side blocks,) about one-sixteenth of an inch square and the thickness of a ten cent piece; drill a hole in each, then solder fast to the plate. Over these the blocks are moulded with holes through the same, over each nut, and two half through, (see fig. F,) to meet what we term steady pins, which are soldered to the plate so as to fit these holes accurately, then cut the screws and thread in the nuts, making a head similar to an ordinary screw; (the hole being counter-sunken) the masticating surface of the block is entirely out of the reach of wear and hindrance, but so as to be readily removed with a common penknife. After having fitted the blocks neatly to the pins and outer band, we then fit an inner band and solder it to the plate, (the blocks being removed for fear of cracking.) This being done and the blocks secured to their plates, the plate is now ready for finishing up.

The advantages of this method are, *first*, *cleanliness*, (which is by no means a trifling consideration,) as the teeth are easily removed from the plate and replaced by the wearer, they can be kept perfectly clean with but little effort. *Second*, the plate is not as liable to warp or spring in soldering, nor to become bent by wearing. *Thirdly*, in case of accident of any kind, they are more readily repaired; for instance, if the plate has been bent or forced out of shape, by any cause, by removing the blocks it can be refitted to a cast much more accurately than if the teeth were soldered, or should a tooth be broken off, a block can be substituted without fear of cracking the others.

The method of giving weight to the lower teeth referred to, (as fig. C,) accomplishes the object aimed at, but as the amount of labor and material is great, it is of necessity much more expensive than the ordinary method, therefore will not be popular. Your readers, as well as yourself, will doubtless remember the efforts made by several dentists to introduce the use of tin as a basis for lower teeth, contending that a coating of gold was sufficient to screen it from the action of the agents to which it would be subjected; but there are few, if any, who now use it in their practice, it having been fairly tested and found wanting. It was this failure that induced the endeavor to obtain the advantages of tin, at the same time avoiding its disadvantages, and in the plan adopted, the end or object is attained, which is as follows: Instead of coating or gilding the tin, we box it up and compel it to do its work unseen and unknown, except to the maker, (unless he communicates to the wearer, which he is in duty and honor bound to do.)

This substitute for tin is made as follows, (see fig. G.) The plate is got up in the usual way of gold but very thin, about 35, with an outer and inner band of the same thickness, the two as far apart as the plate will admit. These bands are made sufficiently wide on exterior high enough to cut out return edges to fold around and between the teeth. This being done, and a correct articulating cast obtained, we then line and hard solder the linings to the teeth, and fit as perfectly as possible, (having previously coated the inside of the plate with muriate of zinc,) warm

FIG. G.

the lower portion of our cast and pour in with a spoon, shaped for the purpose, melted tin sufficient to fill the space unoccupied by the teeth. After coating, the pointed edges of the band are turned over so as to fill between the teeth, and with a hot instrument soldered to the tin. The plate is then burnished down and coated with wax, except between the teeth. In this condition, it is placed in a gilding solution, and by a galvanic battery a thick coat of gold is deposited between the teeth; so should there be any tin exposed, (which, if the plate is nicely fitted, is scarcely possible,) it is thus coated with gold. The wax is then removed, and the whole well polished and again subjected to the gilding process, which gives a pure gold surface to the whole plate or base.

Fig. G represents ten teeth secured in their places and space or sockets for the other 4, with the edges of the outer band cut out to turn over between the teeth.

The case referred to as letter H needs no description, the artist having given a very good representation of it (see fig. H.) My principal reason for making a duplicate of this case was in consequence of its being the first instance met with in our practice where the front teeth and that portion of the jaw had been affected by that disease which renders such substitutes necessary. The patient however informed us in all seriousness that this loss was occasioned by a *fever on the Coast of Africa.*

FIG. H.

The cases referred to as letters F and G are illustrated in cuts J and K. The design or object aimed at in this method is to preserve as much of the tasting surface of the mouth as possible; the tongue comes in contact with that portion of the mouth directly inside the six front teeth, more than any other, it is therefore, the most important part

FIG. J.

to have exposed or left uncovered by the plate; in part of a set as in cut F, we can accomplish it with but little difficulty, providing that portion of the plate which crosses the arch of the mouth is made very strong. As this represents an actual case as before stated we will merely say that the clasp is attached simply to steady the plate, it being retained by the central cavity around the edges of which we soldered a small wire which made the cavity more perfect, and added much towards stiffening that portion of the plate. Entire upper sets (see fig. K) are a little more difficult to adjust; but we have thus far been successful in

FIG. K.

making the plate so as to accomplish the desired object.—The central cavity is formed in the same manner as in cut J, though a different shape. As we cover but a small portion of the mouth with plate it is all-important to have that as strong and firm as possible; it is therefore necessary to get more stiffness to the front portion particularly, than can be obtained in one or two thicknesses of plate. We, therefore, after having the plate perfectly fitted and teeth soldered, cut a strip of gold so as to form an inner band, which is then soldered to the linings of the teeth and inner edge of the plate; this gives sufficient strength and firmness to the plate to bear ordinary pressure.

The teeth referred to as being lined by melting, originated with us seven years since. A short time after having adopted this method, we were informed that the same thing had been done in England and noticed in the American Journals, but as we have not to this day seen the notice referred to, we will give a short description of our "modus operandi."

We first get the exact shape and form desired for the lining by placing a strip of thin platina ribbon or foil, covering the inner surface of the tooth with flux, and the outer surface with plaster, then place gold sufficient to make the desired lining or backing, and melt with the blow-pipe, the gold will follow the platina plate. Where the lining is required to be thicker as at the base, it is done by turning up the edges of the platina.

There are some other peculiarities contained in our contributions to the Fair, but as we have already extended this article far beyond our original intentions, will let this suffice, at least for the present. Yours, truly,

AMBLER & AVERY,
31 Washington Place, New York.

---

## Figures and Tables

**Fig. A—B. f1:**
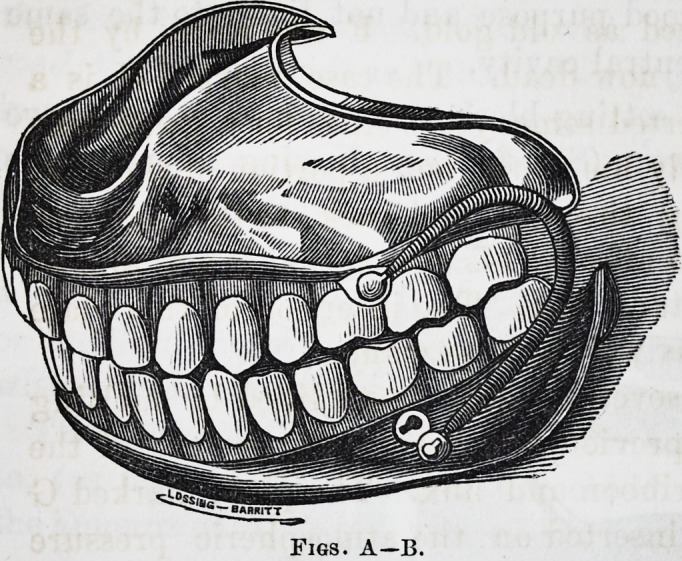


**Fig. C. f2:**
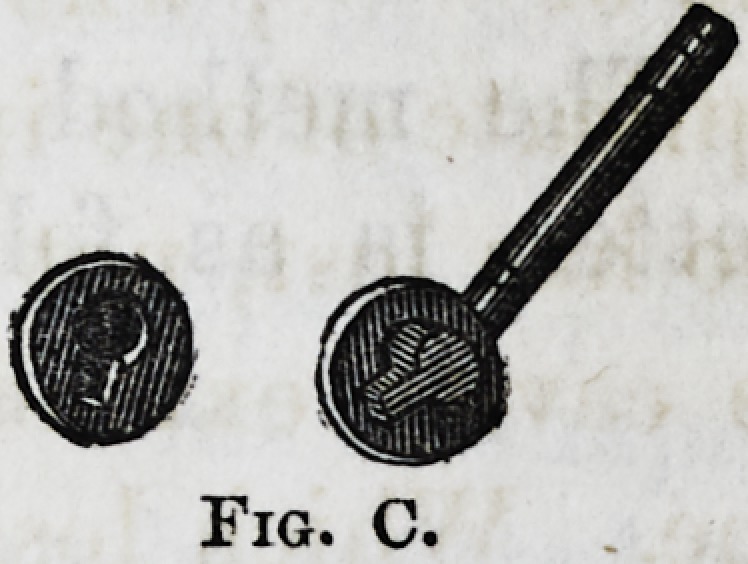


**Fig. D. f3:**
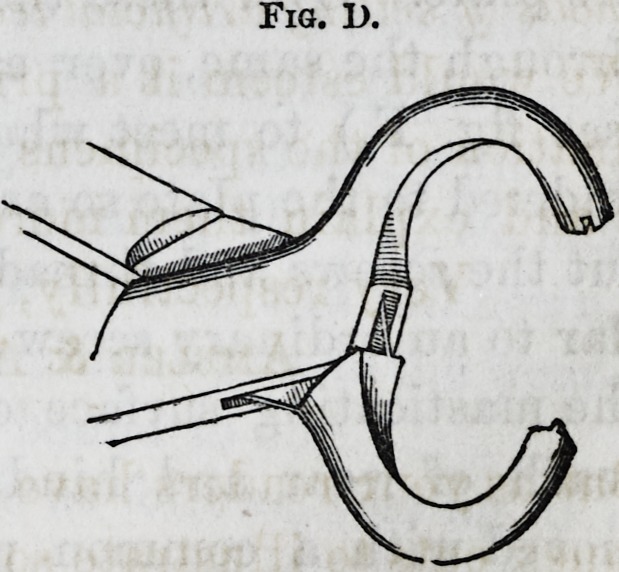


**Figure f4:**
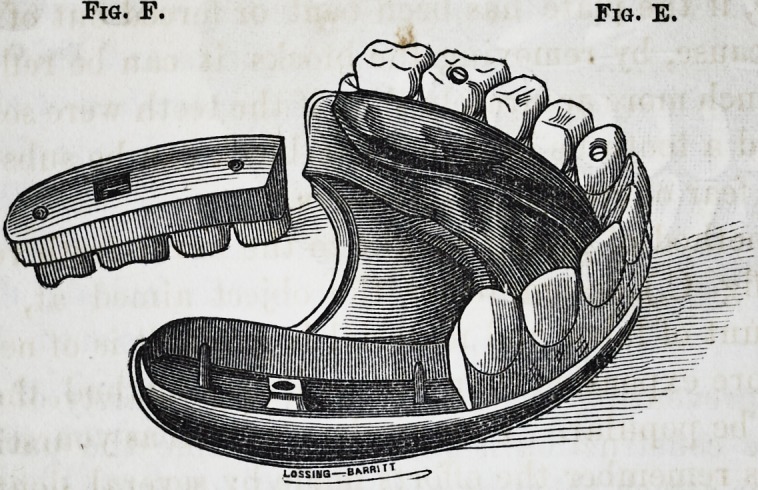


**Fig. G. f5:**
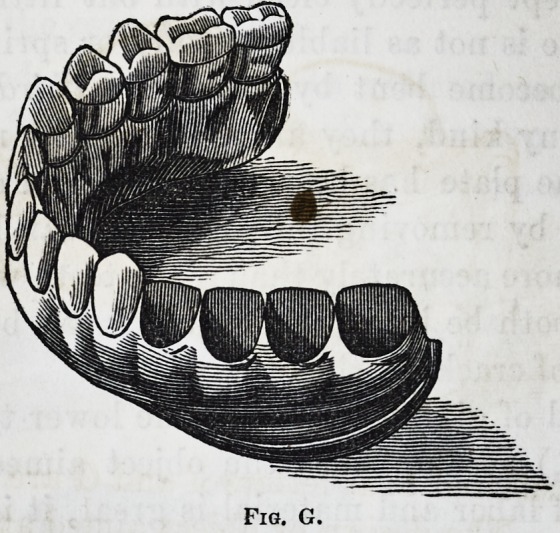


**Fig. H. f6:**
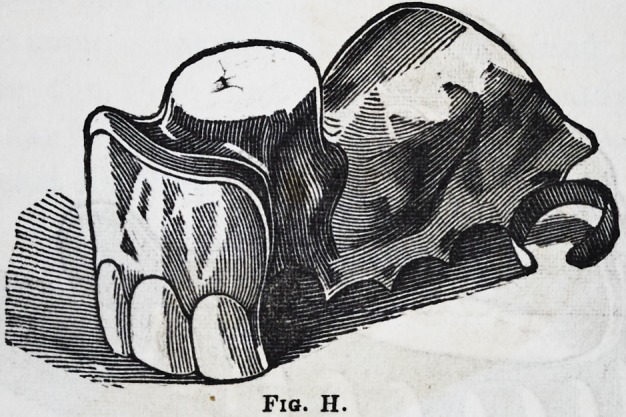


**Fig. J. f7:**
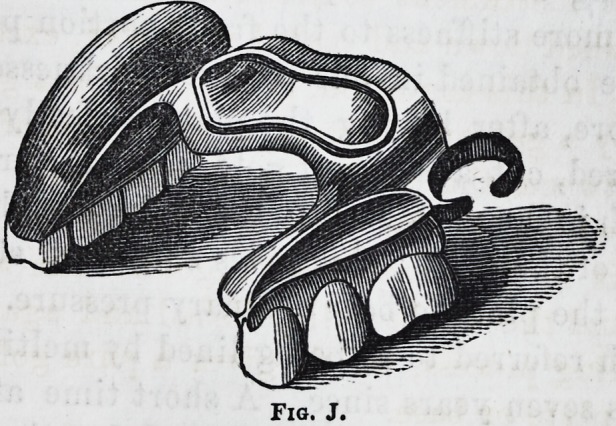


**Fig. K. f8:**